# Daratumumab, carfilzomib, and dexamethasone in relapsed or refractory myeloma: final analysis of PLEIADES and EQUULEUS

**DOI:** 10.1038/s41408-023-00805-x

**Published:** 2023-03-07

**Authors:** Philippe Moreau, Ajai Chari, Albert Oriol, Joaquin Martinez-Lopez, Mathias Haenel, Cyrille Touzeau, Sikander Ailawadhi, Britta Besemer, Javier de la Rubia Comos, Cristina Encinas, Maria-Victoria Mateos, Hans Salwender, Paula Rodriguez-Otero, Cyrille Hulin, Lionel Karlin, Anna Sureda Balari, Joan Bargay, Lotfi Benboubker, Laura Rosiñol, Stefano Tarantolo, Howard Terebelo, Shiyi Yang, Jianping Wang, Ivo Nnane, Ming Qi, Michele Kosh, Maria Delioukina, Hartmut Goldschmidt

**Affiliations:** 1 Hematology, University Hospital Hôtel-Dieu, Nantes, France; 2 Icahn School of Medicine at Mount Sinai, New York, NY USA; 3 Institut Català d’Oncologia and Institut Josep Carreras, Hospital Germans Trias i Pujol, Barcelona, Spain; 4 Hospital 12 de Octubre, H12O-CNIO, Haematological Malignancies Clinical Research Unit, Universidad Complutense, CIBERONC, Madrid, Spain; 5 Klinikum Chemnitz, Chemnitz, Germany; 6 Mayo Clinic Florida, Jacksonville, FL USA; 7 Universitaetsklinikum Tuebingen der Eberhard-Karls-Universitaet, Abteilung fuer Innere Medizin II, Tübingen, Germany; 8 Hospital La Fe, School of Medicine and Dentistry, Catholic University of Valencia, Valencia, Spain; 9 CIBERONC, Instituto Carlos III, Madrid, Spain; 10 Hospital General Universitario Gregorio Marañón (HGUGM), IiSGM, Madrid, Spain; 11 University Hospital of Salamanca/IBSAL/Cancer Research Center-IBMCC (USAL-CSIC), Salamanca, Spain; 12 Asklepios Tumorzentrum Hamburg, AK Altona and AK St. Georg, Hamburg, Germany; 13 Clínica Universidad de Navarra, Pamplona, Spain; 14 Department of Hematology, Hôpital Haut Lévêque, University Hospital, Pessac, France; 15 Department of Hematology, Centre Hospitalier Lyon Sud, Hospices Civils de Lyon, Pierre-Bénite, France; 16 Hematology Department, Institut Català d’Oncologia - Hospitalet, IDIBELL, University of Barcelona, Barcelona, Spain; 17 Hospital Universitari Son Llàtzer, IdISBa, Palma de Mallorca, Illes Balears, Spain; 18 Service d’Hématologie et Thérapie Cellulaire, Hôpital Bretonneau, Centre Hospitalier Régional Universitaire (CHRU), Tours, France; 19 Hospital Clínic de Barcelona, IDIBAPS, Barcelona, Spain; 20 Nebraska Cancer Specialists, Omaha, NE USA; 21 Providence Cancer Center, Southfield, MI USA; 22 Janssen Research & Development, LLC, Spring House, PA USA; 23 GMMG-Study Group at University Hospital Heidelberg, Internal Medicine V, Heidelberg, Germany

**Keywords:** Cancer therapy, Drug therapy

## Introduction

Daratumumab is approved in many countries as monotherapy and in combination regimens for relapsed/refractory multiple myeloma (RRMM) and newly diagnosed multiple myeloma [1–3]. Daratumumab-based combinations have also demonstrated encouraging efficacy in lenalidomide-refractory RRMM [4, 5]. Carfilzomib is approved as monotherapy and in combination regimens, including daratumumab and dexamethasone (D-Kd), for RRMM [6]. In the phase 3 CANDOR study, D-Kd (intravenous [IV] daratumumab; carfilzomib 56 mg/m^2^ twice weekly) improved progression-free survival (PFS) versus Kd in the overall population and lenalidomide-refractory patients [7, 8]. In the phase 3 A.R.R.O.W. study, once-weekly carfilzomib (70 mg/m^2^) significantly prolonged PFS versus twice-weekly carfilzomib (27 mg/m^2^), providing a safe and more convenient Kd dosing regimen [9].

Preliminary results from the phase 2 PLEIADES study (median follow-up, 9.2 months) and the phase 1b EQUULEUS study (median follow-up, 16.6 months) showed that combining subcutaneous daratumumab (DARA SC) and daratumumab IV, respectively, with Kd (carfilzomib 70 mg/m^2^ weekly) was well tolerated and induced deep responses in RRMM patients [10, 11]. We report final data from PLEIADES and EQUULEUS, with a median follow-up of 12.4 and 23.7 months, respectively.

## Methods

PLEIADES (ClinicalTrials.gov Identifier: NCT03412565) evaluated DARA SC (daratumumab 1800 mg co-formulated with recombinant human hyaluronidase PH20 [rHuPH20; 2000 U/mL; ENHANZE^®^ drug delivery technology, Halozyme, Inc., San Diego, CA, USA]) plus weekly Kd (carfilzomib 70 mg/m^2^; dexamethasone 40 mg) in RRMM patients with 1 prior line of lenalidomide-based therapy. EQUULEUS (NCT01998971) evaluated daratumumab IV plus weekly Kd (carfilzomib 70 mg/m^2^; dexamethasone 40 mg) in RRMM patients after 1 to 3 prior lines of therapy (including bortezomib and an immunomodulatory drug). Patients in both studies received 28-day cycles of D-Kd until disease progression. Primary endpoints were the overall response rate (ORR) in PLEIADES and safety and tolerability in EQUULEUS. Supplementary Information includes additional methodology.

## Results

A total of 66 and 85 patients received D-Kd in PLEIADES and EQUULEUS, respectively. Patient demographic and baseline characteristics are summarized in Table S1. In PLEIADES and EQUULEUS, the median (range) age was 61 (42–84) and 66 (38–85) years, respectively, 16/44 (36.4%) and 13/67 (19.4%) patients had high-risk cytogenetics, and 41 (62.1%) and 51 (60.0%) were lenalidomide-refractory. Patients in PLEIADES and EQUULEUS had received a median (range) of 1 (1–1) and 2 (1–4) prior lines of therapy, respectively, and 60 (90.9%) and 85 (100%) patients had received prior proteasome inhibitor therapy. In PLEIADES and EQUULEUS, 31 (47.0%) and 50 (58.8%) patients, respectively, discontinued treatment, primarily due to progressive disease. Median (range) treatment duration was 12.0 (0–21) months in PLEIADES and 19.8 (0.3–34.5) months in EQUULEUS. Patient disposition and drug exposure are further described in Supplementary Information.

Pharmacokinetic data are provided for pharmacokinetic-evaluable populations (PLEIADES, *n* = 65; EQUULEUS, *n* = 85 [single first dose, *n* = 10; split first dose, *n* = 75]). The maximum concentration of DARA SC was observed on Cycle 1, Day 4 after the first dose or Cycle 3, Day 4 after the ninth dose. For daratumumab IV, the maximum concentration was observed on Cycle 1, Day 1 (end of infusion) after the first dose or Cycle 3, Day 1 (end of infusion) after the ninth dose. In both studies, serum trough concentration (C_trough_) increased to maximum C_trough_ on Cycle 3, Day 1 pre-dose, then decreased with less frequent dosing. DARA SC provided numerically higher C_trough_ (within a similar range) versus daratumumab IV. Subgroup analysis of serum daratumumab concentration based on body weight in PLEIADES is presented in Table S2. In EQUULEUS, pharmacokinetic profiles of single and split first daratumumab doses were similar from Cycle 2, Day 1 pre-infusion onward (Table S3). No patient in the immunogenicity-evaluable population of either study tested positive for anti-daratumumab antibodies. In PLEIADES, 3/64 (4.7%) patients in the rHuPH20 immunogenicity-evaluable population had treatment-emergent anti-rHuPH20 antibodies after DARA SC administration; none were neutralizing.

The most common any-grade and grade 3/4 treatment-emergent adverse events (TEAEs) in PLEIADES are summarized in Table 1. Grade 3/4 infections occurred in 9 (13.6%) patients, most commonly pneumonia (4.5%). Serious TEAEs were reported in 22 (33.3%) patients, most commonly pneumonia (4.5%). One (1.5%) patient discontinued treatment due to a TEAE. Three patients had grade 5 TEAEs: 1 each with COVID-19 pneumonia, sepsis, and respiratory failure.

**Table 1:** Most common any-grade (≥25%) and grade 3/4 (≥5%) TEAEs with D-Kd in the PLEIADES and EQUULEUS studies.

	PLEIADES D-Kd (*n* = 66)		EQUULEUS D-Kd (*n* = 85)	
	Any-grade	Grade 3/4	Any grade	Grade 3/4
TEAEs, *n* (%)	66 (100)	49 (74.2)	85 (100)	67 (78.8)
Hematologic				
Thrombocytopenia	34 (51.5)	13 (19.7)	58 (68.2)	27 (31.8)
Anemia	25 (37.9)	8 (12.1)	44 (51.8)	18 (21.2)
Neutropenia	15 (22.7)	7 (10.6)	26 (30.6)	18 (21.2)
Lymphopenia	12 (18.2)	8 (12.1)	25 (29.4)	21 (24.7)
Nonhematologic				
Hypertension	23 (34.8)	14 (21.2)	28 (32.9)	17 (20.0)
Insomnia	23 (34.8)	4 (6.1)	28 (32.9)	4 (4.7)
Diarrhea	20 (30.3)	0	32 (37.6)	2 (2.4)
Nausea	17 (25.8)	0	36 (42.4)	1 (1.2)
Nasopharyngitis	17 (25.8)	0	15 (17.6)	0
Headache	15 (22.7)	0	23 (27.1)	1 (1.2)
Pyrexia	14 (21.2)	1 (1.5)	31 (36.5)	1 (1.2)
Asthenia	14 (21.2)	0	36 (42.4)	13 (15.3)
Cough	13 (19.7)	0	24 (28.2)	0
Dyspnea	12 (18.2)	1 (1.5)	30 (35.3)	3 (3.5)
Upper respiratory tract infection	12 (18.2)	0	38 (44.7)	3 (3.5)
Vomiting	11 (16.7)	0	34 (40.0)	1 (1.2)

*D-Kd* daratumumab/carfilzomib/dexamethasone, *TEAE* treatment-emergent adverse event.

The most common any-grade and grade 3/4 TEAEs in EQUULEUS are summarized in Table 1. Grade 3/4 infections were reported in 18 (21.2%) patients, most commonly pneumonia (4.7%). Serious TEAEs occurred in 41 (48.2%) patients, most commonly basal cell carcinoma, pneumonia, and upper respiratory tract infection (4.7% each). Five (5.9%) patients discontinued treatment due to TEAEs. Three patients had grade 5 TEAEs: two with general physical health deterioration and one with multiple organ dysfunction syndrome.

Two (3.0%) patients in PLEIADES had grade 3/4 cardiac TEAEs: one each with grade 3 cardiac failure and grade 4 left ventricular dysfunction. In EQUULEUS, 9 (10.6%) patients had grade 3/4 cardiac TEAEs, including sinus tachycardia, cardiac failure, systolic dysfunction (*n* = 2 each) and atrial fibrillation, congestive cardiomyopathy, left ventricular failure, myocardial ischemia, and myocarditis (*n* = 1 each). In both studies, the median left ventricular ejection fraction did not notably change over time from baseline. Additional details are included in Supplementary Information.

Three (4.5%) patients had infusion-related reactions (IRRs) with DARA SC in PLEIADES; two had grade 3 IRRs. All patients with IRRs experienced them on the first administration; the median (range) time to IRR onset was 65 (4–75) min. Local injection-site reactions occurred in 7 (10.6%) patients; all were grade 1/2. In EQUULEUS, 6 (60.0%) patients who received a single first dose and 31 (41.3%) who received a split first dose had IRRs with daratumumab IV. Most IRRs were mild (2 patients had grade 3/4 IRRs) and occurred during the first infusion. Five (50.0%) patients experienced IRRs during Cycle 1, Day 1 with a single first daratumumab dose; 27 (36.0%) experienced IRRs during Cycle 1, Day 1 with a split first daratumumab dose.

**Fig. 1:**
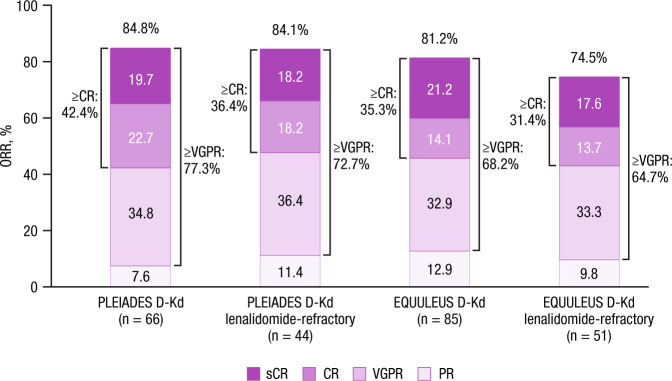
Response rates in all patients and lenalidomide-refractory patients treated with D-Kd in PLEIADES or EQUULEUS. Data were shown for the all-treated population. Note: Due to rounding, percentages may not add up to the total percentage for each category. CR complete response, D-Kd daratumumab/carfilzomib/dexamethasone, ORR overall response rate, PR partial response, sCR stringent complete response, VGPR very good partial response.

In PLEIADES (median [range] follow-up, 12.4 [0.2–20.6] months), ORR was 84.8% in the all-treated population and 84.1% in lenalidomide-refractory patients (Fig. 1). ORR was 75.0% (12/16) and 82.1% (23/28) in patients with high and standard cytogenetic risk, respectively. The median duration of response was not reached; the 9-month duration of response rate was 85.4%. PFS and overall survival (OS) were not analyzed.

In EQUULEUS (median [range] follow-up, 23.7 [0.5–34.7] months), ORR was 81.2% in the all-treated population and 74.5% in lenalidomide-refractory patients (Fig. 1). The median duration of response was 27.5 months; the 9-month duration of response rate was 88.3%. Median PFS was 25.7 months in the all-treated population (Fig. S1A) and 22.3 months in lenalidomide-refractory patients; estimated 24-month PFS rates were 52.7% and 46.9%, respectively. The median time to subsequent anti-cancer therapy was 29.2 months. Median OS was not reached; the estimated 24-month OS rate was 71.2% (Fig. S1B).

## Discussion

With additional follow-up in PLEIADES and EQUULEUS, no new safety concerns were identified, and the overall safety profile of D-Kd was consistent between studies. As previously demonstrated, lower IRR rates and shorter administration times were observed with the DARA SC–based versus daratumumab IV–based regimen. In EQUULEUS, split first daratumumab dosing produced similar safety and pharmacokinetic profiles as a single first dose from Cycle 2, Day 1 pre-infusion onward and decreased median infusion duration on Cycle 1, Day 1, which may improve patient convenience. Following weekly dosing, C_trough_ of daratumumab at Cycle 3, Day 1 pre-dose was numerically higher (within a similar range) with DARA SC versus daratumumab IV. DARA SC administration achieved adequate and consistent exposure for all body weight subgroups. The incidence of treatment-emergent anti-daratumumab antibodies was 0% in both studies, indicating a low risk of immunogenicity to D-Kd. The incidence of treatment-emergent rHuPH20 antibodies was consistent with other DARA SC studies [12, 13].

Final analysis results from both studies showed D-Kd consistently induced deep responses regardless of lenalidomide refractoriness. ORRs in PLEIADES and EQUULEUS were comparable (84.8% and 81.2%, respectively). Encouraging 24-month PFS and OS rates of 52.7 and 71.2%, respectively, were demonstrated in EQUULEUS.

Cross-trial comparisons should be interpreted cautiously due to study differences. The phase 3 CANDOR study [8] compared daratumumab IV plus Kd versus Kd in RRMM patients with 1 to 3 prior lines of therapy. Patients in CANDOR received carfilzomib 56 mg/m^2^ twice weekly (monthly cumulative dose, 336 mg/m^2^ [Cycle 1, 264 mg/m^2^]), while patients in PLEIADES and EQUULEUS received carfilzomib 70 mg/m^2^ weekly (monthly cumulative dose, 210 mg/m^2^ [Cycle 1, 160 mg/m^2^]; ie, 62.5% of CANDOR monthly dose). A higher proportion of patients in PLEIADES and EQUULEUS had prior lenalidomide exposure (100% and 95%, respectively) or were refractory to lenalidomide (62% and 60%) versus the CANDOR D-Kd arm (39% and 32%, respectively). ORRs in PLEIADES (84.8%) and EQUULEUS (81.2%) were comparable to that reported at a median follow-up of 27.8 months in the CANDOR D-Kd arm (84%), with higher complete response or better rates (42.4% and 35.3% vs 33%, respectively). Median PFS was similar between the D-Kd arms of EQUULEUS and CANDOR (25.7 and 28.6 months, respectively) [8]. Overall, D-Kd demonstrated efficacy in PLEIADES, EQUULEUS, and CANDOR, supporting its use for RRMM, including lenalidomide-refractory multiple myeloma. Safety profiles were generally similar with D-Kd utilizing weekly carfilzomib dosing in PLEIADES and EQUULEUS and twice-weekly carfilzomib dosing in CANDOR; however, the incidence of fatal TEAEs was lower in PLEIADES (3/66 [4.5%]) and EQUULEUS (3/85 [3.5%]) compared to the CANDOR D-Kd arm (27/308 [8.8%]) [8]. Results from the phase 3 A.R.R.O.W. study also reinforce the favorable benefit-risk profile for once-weekly carfilzomib dosing compared with a twice-weekly treatment schedule [9]. Once-weekly carfilzomib dosing may improve cost-effectiveness and convenience for patients and healthcare providers [14].

Limitations of PLEIADES and EQUULEUS include small sample sizes and lack of comparator arms. Also, patients in PLEIADES were only followed for up to 8 weeks after the last study treatment; therefore, PFS and OS were not evaluated.

In conclusion, D-Kd continues to be well tolerated and effective in RRMM patients, including lenalidomide-refractory patients. Final analysis results from PLEIADES and EQUULEUS further support D-Kd as a standard treatment regimen for RRMM.

## Supplementary information

Supplemental Appendix

## Data Availability

The data sharing policy of Janssen Pharmaceutical Companies of Johnson & Johnson is available at https://www.janssen.com/clinical-trials/transparency. As noted on this site, requests for access to the study data can be submitted through Yale Open Data Access (YODA) Project site at http://yoda.yale.edu.
