# Supplementary material for: Daratumumab, carfilzomib, and dexamethasone in relapsed or refractory myeloma: final analysis of PLEIADES and EQUULEUS
Source: Blood Cancer J. 2023 Mar 7;13(1):33. doi: 10.1038/s41408-023-00805-x (PMC9989580; doi:10.1038/s41408-023-00805-x)
Supplement: Supplementary file 1 — Supplemental Appendix [file 41408_2023_805_MOESM1_ESM.docx]

# Supplementary Information for Moreau et al. Daratumumab, carfilzomib, and dexamethasone in relapsed or refractory myeloma: final analysis of PLEIADES and EQUULEUS

## **Supplementary Methods**

PLEIADES (ClinicalTrials.gov Identifier: NCT03412565) is a multicenter, open-label, phase 2 study of subcutaneous daratumumab (DARA SC) plus a variety of regimens, including carfilzomib (70 mg/m^2^ weekly) and dexamethasone (D-Kd) in relapsed or refractory multiple myeloma patients with 1 prior line of lenalidomide-based therapy. EQUULEUS (ClinicalTrials.gov Identifier: NCT01998971) is a multicenter, open-label phase 1b study evaluating daratumumab intravenous plus a variety of regimens, including D-Kd (carfilzomib 70 mg/m^2^ weekly) in relapsed or refractory multiple myeloma patients after 1–3 prior lines of therapy (including bortezomib and an immunomodulatory drug). Study protocols and amendments were approved by the institutional review board or independent ethics committee at each site. All patients gave written informed consent. Both studies were conducted per the International Conference on Harmonization Good Clinical Practice guidelines, the principles originating from the Declaration of Helsinki, and study site–specific regulations.

### *Participants*

In PLEIADES and EQUULEUS, eligible patients were aged ≥18 years, had relapsed or refractory multiple myeloma according to International Myeloma Working Group criteria [1], and had an Eastern Cooperative Oncology Group performance status score ≤2. Patients in PLEIADES received 1 prior line of therapy (including ≥2 lenalidomide cycles), achieved at least partial response (PR) to the first treatment regimen, and progressed from or were refractory to their first line of treatment. Patients in EQUULEUS received 1–3 prior lines of therapy (including bortezomib and an immunomodulatory drug), achieved ≥PR to 1 prior line of therapy, and had disease progression after their last line of therapy. Lenalidomide-refractory patients whose disease progressed after their last therapy (or within 60 days of completion of any lenalidomide dose) were allowed. In both studies, eligible patients had left ventricular ejection fraction (LVEF) ≥40%, absolute neutrophil count ≥1.0×10^9^/L, platelet count ≥75×10^9^/L, and creatinine clearance ≥20 mL/min. Patients were excluded if they received previous treatment with daratumumab (or other anti-CD38 therapies) or carfilzomib, had undergone autologous stem cell transplant within 12 weeks before treatment initiation, had received anti-myeloma therapy within 2 weeks before starting treatment, or had grade ≥3 neuropathy or neuropathic pain (PLEIADES only). Patients with meningeal involvement of multiple myeloma; chronic obstructive pulmonary disease (forced expiratory volume in 1 second <50% of predicted normal); moderate, severe, or uncontrolled asthma; or significant heart disease were excluded.

### *Study design and treatment*

In PLEIADES, DARA SC was administered weekly (Days 1, 8, 15, and 22) during Cycles 1 and 2, every 2 weeks (Days 1 and 15) during Cycles 3–6, and every 4 weeks thereafter. In EQUULEUS, daratumumab intravenous (16 mg/kg) was administered weekly (Days 1, 8, 15, and 22) during Cycles 1 and 2, every 2 weeks (Days 1 and 15) during Cycles 3–6, and every 4 weeks thereafter. Ten patients received a single first daratumumab dose (16 mg/kg) on Day 1 of Cycle 1, while remaining patients received the first dose split over 2 days (8 mg/kg on Days 1 and 2 of Cycle 1). In both studies, carfilzomib was administered weekly on Days 1, 8, and 15 of each 28-day cycle. Patients received an initial carfilzomib dose of 20 mg/m^2^ on Cycle 1 Day 1, which increased to 70 mg/m^2^ on Cycle 1 Day 8 onwards, if tolerated. Dexamethasone was administered at 40 mg/week (20 mg/week in patients aged >75 years). During daratumumab treatment weeks, dexamethasone (20 mg) was administered immediately before infusion and the day after infusion; during weeks when patients did not receive daratumumab, dexamethasone was administered as a single 40 mg dose.

Pre-infusion medications included diphenhydramine, acetaminophen, and dexamethasone; montelukast was required before the first dose (EQUULEUS only) and was optional for subsequent doses.

### *Outcomes*

In PLEIADES, secondary endpoints included very good partial response or better rate, complete response or better rate, duration of response (defined as the time from initial documented response [≥PR] to first documented evidence of progressive disease or death due to progressive disease), and infusion-related reaction rate. In EQUULEUS, secondary endpoints were ORR and overall survival; exploratory endpoints included progression-free survival and pharmacokinetics. Safety evaluations included adverse event (AE) monitoring, physical examinations, electrocardiogram and echocardiogram monitoring, clinical laboratory tests, vital sign measurements, and Eastern Cooperative Oncology Group performance status score. In PLEIADES, patients were followed for up to 8 weeks after the last dose of study treatment (data cutoff: 7 December 2020); in EQUULEUS, patients were followed until patient withdrawal, death, or end of study (data cutoff: 31 January 2019).

### *Statistical analyses and assessments*

Samples for pharmacokinetic analysis were collected pre-dose on Day 1 of Cycles 1, 3, 6, 9, and 12, post-infusion on Day 4 of Cycles 1 and 3, and at post-treatment visits 30 days and 8 weeks after the last dose in PLEIADES and pre-dose and post-infusion on Day 1 of Cycles 1–4 (and Day 2 Cycle 1 for patients receiving a split first dose) and Weeks 3 and 9 of the follow-up phase in EQUULEUS; samples were analyzed as described previously [2]. Samples for immunogenicity analysis were collected pre-dose on Day 1 of Cycles 1, 3, 6, 9, and 12 and at post-treatment visits 30 days and 8 weeks after last dose in PLEIADES (daratumumab and rHuPH20) and pre-dose on Day 1 of Cycle 1 and at Weeks 3 and 9 of the follow-up phase in EQUULEUS (daratumumab only).

Toxicities were graded using the National Cancer Institute Common Terminology Criteria for Adverse Events Version 4 [3]. Treatment-emergent AEs were summarized using descriptive statistics, including AEs of interest.

Response and disease progression were assessed by a computerized algorithm based on International Myeloma Working Group consensus criteria [4, 5]. Progression-free survival was estimated using the Kaplan–Meier method for descriptive summaries. In both studies, high cytogenetic risk was defined as the presence of del(17p), t(4;14), or t(14;16) abnormalities and was assessed by fluorescence in situ hybridization or karyotype testing.

## **Supplementary Results**

### *Patient disposition and drug exposure*

Of the 66 patients in PLEIADES, 31 (47.0%) patients discontinued treatment due to progressive disease (24 [36.4%]), death (3 [4.5%], including 1 caused by COVID-19), patient withdrawal (2 [3.0%]), AE (1 [1.5%]), and other reasons (1 [1.5%]). Of the 85 patients in EQUULEUS, 50 (58.8%) patients discontinued treatment due to progressive disease (36 [42.4%]), patient withdrawal (6 [7.1%]), AE (5 [5.9%]), physician decision (2 [2.4%]), and death (1 [1.2%]).

Patients received a median (range) 13.0 (1–23) D-Kd cycles in PLEIADES and 21.0 (1–37) D-Kd cycles in EQUULEUS. Median (range) relative dose intensity was 100% (75–100) for DARA SC, 94.6% (48–102) for carfilzomib, and 86.6% (43–101) for dexamethasone in PLEIADES and 99.8% (49–108) for daratumumab intravenous, 95.0% (22–105) for carfilzomib, and 97.9% (50–101) for dexamethasone in EQUULEUS. Median duration of administration of DARA SC in PLEIADES was 5 minutes during the first, second, and subsequent administrations. In EQUULEUS, median (range) duration of infusion for the single first daratumumab dose on Cycle 1 Day 1 (n = 9) was 7.1 (6.5–8.9) hours; median (range) duration of infusion for the split first daratumumab dose (n = 75) was 4.3 (3.9–10.6) hours on Cycle 1 Day 1 and 4.2 (3.9–8.6) hours on Cycle 1 Day 2. Median (range) durations of infusion were similar for all subsequent infusions in patients who received single (3.4 [2.5–5.6] hours) and split (3.4 [2.3–5.9] hours) first daratumumab doses.

### *Pharmacokinetics*

Subgroup analysis of serum daratumumab concentration based on body weight among patients in PLEIADES is presented in **Table S2**. For the lowest body weight subgroup (≤65 kg), observed mean maximum serum trough concentration (C_trough_) at Cycle 3 Day 1 was 27.8% higher than that of the total pharmacokinetic-evaluable population. For the >65–85 kg body weight subgroup, mean maximum C_trough_ at Cycle 3 Day 1 was comparable to that of the total pharmacokinetic-evaluable population. For the highest body weight subgroup (>85 kg), mean maximum C_trough_ at Cycle 3 Day 1 was 31.6% lower than that of the total pharmacokinetic-evaluable population.

### *Safety*

In both studies, median LVEF was not notably changed from baseline over time overall. In PLEIADES, median (range) LVEF was 61% (41–78) at baseline (n = 66), 60% (30–80) at Month 6 (n = 40), and 61% (50–74) at Month 12 (n = 36). In EQUULEUS, median (range) LVEF was 64% (44–83) at baseline (n = 84), 62% (46–77) at Cycle 6 (n = 54), 61% (32–76) at Cycle 12 (n = 47), 59% (50–74) at Cycle 18 (n = 22), and 63% (53–76) at Cycle 24 (n = 10). Diastolic dysfunction and global longitudinal strain were not reported in either study.

# Supplementary Table S1. Patient demographic and baseline disease characteristics and prior multiple myeloma therapies received in the PLEIADES and EQUULEUS D-Kd studies.

|  | **PLEIADES  D-Kd**  **All treated  (n = 66)** | **EQUULEUS  D-Kd**  **All treated**  **(n = 85)** |
| --- | --- | --- |
| Age, median (range), years  ≥75, n (%) | 61 (42–84)  4 (6.1) | 66 (38–85)  8 (9.4) |
| Male, n (%) | 34 (51.5) | 46 (54.1) |
| Body weight, median (range), kg | 73.7 (48.0–113.9) | 70.0 (45.0–160.8) |
| Race, White, n (%) | 48 (72.7) | 68 (80.0) |
| ECOG PS score, n (%)  0  1  2 | 40 (60.6)  23 (34.8)  3 (4.5) | 32 (37.6)  46 (54.1)  7 (8.2) |
| ISS disease stage, n (%)  I  II  III | 45 (68.2)  12 (18.2)  9 (13.6) | NA  NA  NA |
| Prior lines of therapy, n (%)  Median (range)  1  2  3  >3 | 1 (1–1)  66 (100)  0  0  0 | 2 (1–4)  20 (23.5)  40 (47.1)  23 (27.1)  2 (2.4)^a^ |
| Prior ASCT, n (%) | 52 (78.8) | 62 (72.9) |
| Prior PI, n (%)  Bortezomib  Ixazomib | 60 (90.9)  58 (87.9)  7 (10.6) | 85 (100)  85 (100)  8 (9.4) |
| Prior IMiD, n (%)  Lenalidomide  Pomalidomide  Thalidomide | 66 (100)  66 (100)  0  11 (16.7) | 85 (100)  81 (95.3)  13 (15.3)  21 (24.7) |
| Prior PI + IMiD, n (%) | 60 (90.9) | 85 (100) |
| Prior PI + IMiD + ALKY, n (%) | 54 (81.8) | 81 (95.3) |
| Refractory to,^b^ n (%)  Lenalidomide  Pomalidomide  Bortezomib  PI + IMiD  Last prior line of therapy | 41 (62.1)  0  5 (7.6)  9 (13.6)  41 (62.1) | 51 (60.0)  11 (12.9)  26 (30.6)  25 (29.4)  54 (63.5) |
| Type of measurable disease, n (%)  Serum and urine  IgG  IgA  Other  Serum only  IgG  IgA  Other  Urine only  Serum FLC only | 5 (7.6)  NA  NA  NA  35 (53.0)  28 (42.4)  6 (9.1)  1 (1.5)  10 (15.2)  16 (24.2) | 15 (17.6)  10 (11.8)  4 (4.7)  1 (1.2)  37 (43.5)  28 (32.9)  8 (9.4)  1 (1.2)  19 (22.4)  14 (16.5) |
| Times since initial diagnosis of multiple myeloma, median (range), months | 32.3 (6.9–132.2) | 49.7 (9.0–145.9) |
| Bone marrow % plasma cells, n (%)  n  <10  10–30  >30 | 65  21 (32.3)  23 (35.4)  21 (32.3) | 85  22 (25.9)  29 (34.1)  34 (40.0) |
| Cytogenetic profile,^c^ n (%)  n  High risk  Standard risk | 44  16 (36.4)  28 (63.6) | 67  13 (19.4)  54 (80.6) |

Safety population, defined as patients who received ≥1 dose of study treatment.

ALKY, alkylator; ASCT, autologous stem cell transplant; D-Kd, daratumumab/carfilzomib/dexamethasone; ECOG PS, Eastern Cooperative Oncology Group performance status; FLC, free light chain; IgA, immunoglobulin A; IgG, immunoglobulin G; IMiD, immunomodulatory drug; ISS, International Staging System; NA, not available; PI, proteasome inhibitor.

^a^Two patients received 4 prior lines of therapy and were considered protocol deviations.

^b^Refractoriness was based on the most recent prior medication.

^c^Based on fluorescence in situ hybridization/karyotype testing.

# Supplementary Table S2. PLEIADES: DARA SC serum concentrations by body weight.

|  | **DARA SC serum concentrations, µg/mL** | | | |
| --- | --- | --- | --- | --- |
|  |  | **Body weight subgroups** | | |
|  | **Total** | **≤65 kg** | **>65**–**85 kg** | **>85 kg** |
| Cycle 1 Day 4 post-dose, n  Mean (SD) | 57  137 (56.7) | 15  167 (64.7) | 27  135 (51.8) | 15  111 (44.9) |
| Cycle 3 Day 1 pre-dose, n  Mean (SD) | 60  744 (289) | 16  951 (255) | 28  759 (240) | 16  509 (235) |
| Cycle 3 Day 4 post-dose, n  Mean (SD) | 46  853 (292) | 13  1055 (209) | 18  884 (231) | 15  641 (292) |
| Cycle 6 Day 1 pre-dose, n  Mean (SD) | 49  728 (275) | 12  842 (346) | 23  791 (224) | 14  528 (175) |
| Cycle 9 Day 1 pre-dose, n  Mean (SD) | 41  577 (238) | 11  669 (242) | 23  594 (235) | 7  373 (109) |
| Cycle 12 Day 1 pre-dose, n  Mean (SD) | 38  459 (194) | 10  547 (172) | 21  483 (191) | 7  263 (75.6) |
| End of treatment, n  Mean (SD) | 13  247 (228) | 6  363 (235) | 3  40.7 (37.6) | 4  228 (213) |

All Cycle 1 Day 1 pre-dose daratumumab concentrations were below the lower limit of quantitation.

DARA SC, subcutaneous daratumumab; SD, standard deviation.

# Supplementary Table S3. EQUULEUS: daratumumab IV serum concentration with single versus split first dose.

|  | **Daratumumab IV serum concentrations, µg/mL** | | |
| --- | --- | --- | --- |
|  | **Single first dose** | **Split first dose** | **Total** |
| Cycle 1 Day 1 post-infusion, n  Mean (SD) | 8  321 (49) | 71  156 (49) | 79  173 (70) |
| Cycle 1 Day 2 pre-infusion, n  Mean (SD) | –  – | 65  113 (43) | 65  113 (43) |
| Cycle 1 Day 2 post-infusion, n  Mean (SD) | –  – | 69  255 (72) | 69  255 (72) |
| Cycle 2 Day 1 pre-infusion, n  Mean (SD) | 10  332 (115) | 63  363 (179) | 73  359 (172) |
| Cycle 2 Day 1 post-infusion, n  Mean (SD) | 9  690 (152) | 65  702 (254) | 74  700 (243) |
| Cycle 3 Day 1 pre-infusion, n  Mean (SD) | 9  517 (137) | 52  619 (256) | 61  604 (244) |
| Cycle 3 Day 1 post-infusion, n  Mean (SD) | 9  896 (170) | 52  951 (350) | 61  943 (329) |
| Cycle 4 Day 1 pre-infusion, n  Mean (SD) | 7  515 (159) | 55  570 (242) | 62  564 (234) |
| Cycle 4 Day 1 post-infusion, n  Mean (SD) | 8  912 (175) | 55  951 (295) | 63  946 (282) |

All Cycle 1 Day 1 pre-dose daratumumab concentrations were below the lower limit of quantitation.

IV, intravenous; SD, standard deviation.

# Supplementary Figure S1. (A) PFS and (B) OS with D-Kd in all-treated patients in EQUULEUS.


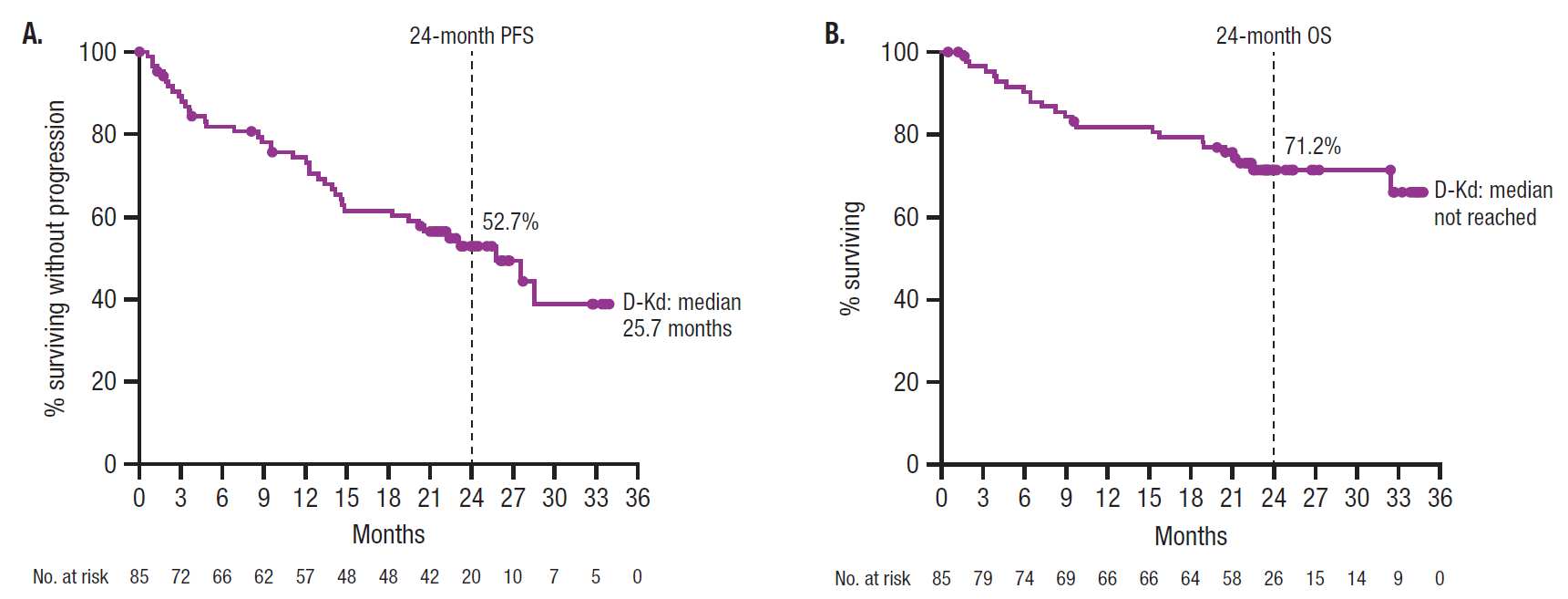


D-Kd, daratumumab/carfilzomib/dexamethasone; OS, overall survival; PFS, progression-free survival.

# Supplementary References

1. Rajkumar SV, Dimopoulos MA, Palumbo A, Blade J, Merlini G, Mateos MV, et al. International Myeloma Working Group updated criteria for the diagnosis of multiple myeloma. Lancet Oncol. 2014; 15:e538–e548.

2. Clemens PL, Yan X, Lokhorst HM, Lonial S, Losic N, Khan I, et al. Pharmacokinetics of daratumumab following intravenous infusion in relapsed or refractory multiple myeloma after prior proteasome inhibitor and immunomodulatory drug treatment. Clin Pharmacokinet. 2017; 56:915–924.

3. US Department of Health and Human Sercives, National Institutes of Health, National Cancer Institute. Common Terminology Criteria for Adverse Events (CTCAE) Version 4.03. 2010. <https://evs.nci.nih.gov/ftp1/CTCAE/CTCAE_4.03/CTCAE_4.03_2010-06-14_QuickReference_5x7.pdf>. Accessed 15 Jun 2022.

4. Durie BG, Harousseau JL, Miguel JS, Bladé J, Barlogie B, Anderson K, et al. International uniform response criteria for multiple myeloma. Leukemia. 2006; 20:1467–1473.

5. Rajkumar SV, Harousseau JL, Durie B, Anderson KC, Dimopoulos M, Kyle R, et al. Consensus recommendations for the uniform reporting of clinical trials: report of the International Myeloma Workshop Consensus Panel 1. Blood. 2011; 117:4691–4695.
